# Long-term psychological distress of Bosnian war survivors: an 11-year follow-up of former displaced persons, returnees, and stayers

**DOI:** 10.1186/s12888-018-1996-0

**Published:** 2019-01-03

**Authors:** Hannah Comtesse, Steve Powell, Andrea Soldo, Maria Hagl, Rita Rosner

**Affiliations:** 1 Department of Psychology, Catholic University Eichstaett-Ingolstadt, Ostenstrasse 25, 85071 Eichstaett, Germany; 2 proMENTE social research, Sarajevo, Bosnia and Herzegovina; 3 Munich, Germany

**Keywords:** War trauma, Displacement, Long-term mental health, Brief symptom inventory (BSI), Bosnia and Herzegovina

## Abstract

**Background:**

Research on the long-term mental health consequences of war and displacement among civilians who live in post-conflict countries is rare. The aim of this study was to examine the developmental trajectories and predictors of general psychological distress in three samples of Bosnian war survivors over an 11-year period.

**Methods:**

In 1998/99, about three years after the war in Bosnia and Herzegovina, a representative sample of 299 adult Sarajevo citizens was examined in three subsamples: individuals who had stayed in Sarajevo throughout the siege, individuals who had been internally displaced, and refugees who had returned. Of the 138 study participants who could be located 11 years later, 100 were re-assessed (71%) using the Brief Symptom Inventory.

**Results:**

Over time, psychological symptoms and general psychological distress decreased in those survivors who had stayed and increased in returnees. Former displaced persons did not show any significant changes. After controlling for other factors, cumulative trauma exposure before and during the war predicted general psychological distress at baseline. Eleven years later, higher trauma exposure during and after the war, returnee status, and more current stressors were all associated with higher levels of general psychological distress.

**Conclusions:**

Levels of psychological symptoms remained high in three subsamples of Bosnian war survivors. The differential symptom trajectories may correspond to distinct war experiences and contemporary stressors. Still, the cumulative effect of war traumata on mental distress persisted more than a decade after war and displacement, although the influence of current stressors seemed to increase over time.

**Electronic supplementary material:**

The online version of this article (10.1186/s12888-018-1996-0) contains supplementary material, which is available to authorized users.

## Background

### Mental Health Research after war and displacement

The current migrant crisis is unprecedented in modern Europe. In 2016, Europe experienced the largest influx of forcibly displaced people since the Second World War [1]. At the same time, the number of returnees increased globally, particularly to low- and middle-income countries [1]. Therefore, it is important to evaluate the long-term impact of war traumatization on the mental health of civilians living in post-conflict regions in order to inform policies for long-term rehabilitation. However, there is a dearth of longitudinal studies. Most studies were short-term follow-ups (up to three years) conducted in the immediate post-conflict/post-migration period and were not able to distinguish between long-term and temporary psychological distress [2]. The few longer-term follow-up studies (up to ten years) were limited to specific psychological symptoms or high-income Western settings [3–5]. Moreover, the majority of studies focused on a small set of psychological symptoms or syndromes, mostly depression and PTSD. This study is the longest follow-up conducted on the course and predictors of general mental health of people affected by the war in Bosnia and Herzegovina (BiH) from 1992 to 1995 who live in the post-conflict region.

### Long-term mental health consequences of the Yugoslav wars

Cross-sectional research on the mental health consequences of the war in BiH clearly attests to its often devastating effects [6–9]. Priebe and colleagues [8] observed prevalence rates of 22% for mood disorders and 35% for post-traumatic stress disorder (PTSD) in Bosnian citizens on average 11 years after the war in BiH. Moreover, general psychological symptom levels were higher than those usually observed in Western general populations [8]. Meta-analyses suggested several predictors of less favorable mental health outcomes after war and displacement, including older age, female gender, higher education, shorter time since conflict, higher trauma exposure, displacement, restricted economic opportunities, and ongoing political conflict [6–8]. Furthermore, previous research has shown that current stressors and living difficulties (e.g., restricted economic opportunities, social isolation) have a negative impact on mental health beyond the influence of cumulative trauma exposure [7, 10]. In a 3-year follow-up on Bosnian refugees in Croatia, about four years after the war, Mollica et al. [11] found that 43% of those who met the criteria for depression (alone or comorbid with PTSD) at baseline continued to do so, and that an additional 16% had become symptomatic. In a 1.5-year follow-up on a sample of Kosovar Albanian refugees in Sweden, PTSD rates and depression symptoms increased, and even more so in asylum seekers than in those who had returned to Kosovo at follow-up [12, 13]. In contrast, PTSD rates and self-reported mental health improved over a 6-year follow-up period in another sample of war-affected Kosovar Albanians [14]. Proroković and colleagues [5] monitored three samples of Croatian men (civilians, refugees, and soldiers) over a nine-year period after the war. The groups showed different symptom trajectories: somatic complaints increased in soldiers but mostly remained the same in male civilians. Although refugees initially displayed the highest levels of depressive and somatic symptoms, their symptom levels decreased over time. Overall, psychological symptom levels seem to increase in the early post-conflict period and then decline over the ensuing years.

### Study goal

In 1998 and 1999, three to four years after the war in BiH, we conducted several surveys attesting to the high general psychological symptom levels of Sarajevo citizens [15–18]. The aim of this study was to follow up three samples of Sarajevo residents eleven years later on general mental health. The samples encompassed persons who had stayed in Sarajevo throughout the siege, former internally displaced persons, and returnees who had sought refuge in other countries during the war. Based on previous research, we expected decreases in psychological symptom levels over the 11-year follow-up period in each group. We also examined lifetime trauma exposure and current stress factors as predictors of general psychological distress.

## Method

### Setting and participants

The baseline assessment had been conducted in Sarajevo in 1998 and 1999 as part of a larger study in BiH [15–18]. Sarajevo was besieged by Bosnian Serb forces throughout the entire Bosnian war from 1992 to 1995. Civilians could not leave the city for most of this period, and the entire infrastructure and most of the buildings were severely damaged [19].

A random sampling approach was used to recruit three samples that were representative of the prewar Bosnian general population [15–18]. Stratification by age and gender was based on prewar census data for former Yugoslavia. The overall inclusion criteria were: (a) age between 16 and 65 years, (b) resident in Sarajevo at time of assessment, (c) living in former Yugoslavia for most of 1980 to 1991, (d) no psychotic disorder or acute crisis, and (e) being literate enough in Bosnian, Croatian, or Serbian in order to answer questionnaires with help from interviewers. The first sample was recruited from persons living in randomly selected streets and consisted of 98 “stayers” who had lived in Sarajevo throughout the siege (residents sample in Rosner et al. [18]). Samples 2 and 3 were recruited from persons who had been forcibly displaced for more than 12 months between 1992 and 1995 (see Powell et al. [17]). Participants were randomly selected from lists held at a randomized selection of local councils in Sarajevo in 1999. Sample 2, “displaced persons”, consisted of 97 individuals living in Sarajevo who had been internally displaced to other countries in former Yugoslavia. The third sample, “returnees”, included 104 persons living in Sarajevo who had taken refuge in countries outside former Yugoslavia (71% in Germany). Deadline to return or expulsion (50%), homesickness (73%), and reuniting with family members (52%) were the reasons most frequently given for returning to Sarajevo.

Tables 1 and 2 present demographic data and information on war experiences. All participants reported having experienced events during war that would be considered as traumatic according to the current PTSD definition [20].

**Table 1:** Sociodemographic and trauma characteristics

Characteristic	Total sample (*N* = 98)	
	Baseline	Follow-up
Female, % (*n*)	62.2 (61)	62.2 (61)
Mean age (*SD*)	36.5 (12.1)	47.5 (12.2)
Education, % (*n*)		
Primary	20.4 (20)	15.3 (15)
Secondary	65.3 (64)	60.2 (59)
Tertiary	12.2 (12)	23.5 (23)
Marital status, % (*n*)		
Married/long-term relationship	68.4 (67)	66.3 (65)
Single/divorced/separated/widowed	31.6 (31)	33.7 (33)
Employment status, % (*n*)		
Employed	26.5 (26)	51.0 (50)
Unemployed	35.7 (35)	15.3 (15)
Retired	8.2 (8)	16.3 (16)
In training/education	10.2 (10)	1.0 (1)
Other	19.3 (19)	14.2 (14)
Monthly income, % (*n*)		
No income	16.2 (16)	23.5 (23)
< 500 KM	36.7 (36)	22.4 (22)
500–1000 KM	6.1 (6)	32.7 (32)
> 1000 KM	4.0 (4)	18.4 (18)
Mental health care use, % (*n*)^a^	0.0 (0)	12.2 (12)
Number of traumatic events, mean (*SD*)		
Prewar traumatic events	1.01 (1.93)	0.34 (0.78)
Traumatic events during the war	19.54 (11.58)^b^	2.38 (2.20)^c^
Postwar traumatic events	–	0.48 (0.96)
Number of current stressors, mean (*SD*)	2.48 (2.47)^d^	2.26 (1.82)^e^

*Note*: (−) not calculated, *KM* = “convertible Marks”. ^a^ Includes work with psychiatrists, psychologists, or social workers in the previous three months. ^b^ Traumatic events assessed with the CWE (Rosner et al. [18]; range: 0–98). ^c^ Adapted trauma list of the PDS (Foa et al. [25]; range: 0–13). ^d^ 23-item stressor list based on the CWE (range: 0–23). *N* = 65 as the checklist was not completed by stayers. ^e^ 12-item stressor list based on the CWE (range: 0–12)

**Table 2:** War-related characteristics and types of current stressors

	Stayers (*n* = 33)		Displaced (*n* = 27)		Returnee (*n* = 38)	
	Baseline	Follow-up	Baseline	Follow-up	Baseline	Follow-up
Number of years in war zone, mean (*SD*)	–		2.93 (1.88)		1.99 (1.73)	
Number of years in displacement setting, mean (*SD*)	–		0.95 (1.87)		4.21 (1.59)	
Loss of possessions, % (*n*)	39.3 (13)		81.40 (22)		68.4 (26)	
Damage to home, % (*n*)	54.5 (18)		48.10 (13)		78.9 (30)	
Repatriation-related variables, mean (*SD*)^a^						
Material satisfaction	–		−0.01 (1.05)		−0.19 (1.12)	
Identification with home country	–		0.11 (0.57)		0.86 (0.59)	
Community feeling	–		0.51 (1.01)		−0.67 (1.19)	
Type of current stressor, % (*n*)^bc^						
Bad living conditions	–	27.2 (9)	59.2 (16)	18.5 (5)	52.6 (20)	21.1 (8)
Worries about stay in accommodation	–	9.1 (3)	74.1 (20)	11.1 (3)	28.9 (11)	13.1 (5)
Lack of identity papers	–	12.1 (4)	14.8 (4)	7.4 (2)	7.8 (3)	10.5 (4)
Unemployment	–	15.1 (5)	25.9 (7)	40.7 (11)	36.8 (14)	13.1 (5)
Bad job conditions	–	18.2 (6)	14.8 (4)	14.8 (4)	13.1 (5)	10.5 (4)
Little help with welfare	–	18.2 (6)	7.4 (2)	18.5 (5)	10.5 (4)	18.4 (7)
Debts	–	12.1 (4)	48.1 (13)	11.1 (3)	47.3 (18)	15.7 (6)
Serious health problems	–	48.4 (16)	44.4 (12)	55.5 (15)	39.4 (18)	36.8 (14)
Poor access to medical care	–	24.2 (8)	22.2 (6)	37.1 (10)	15.7 (6)	10.5 (4)
Family problems	–	9.1 (3)	11.1 (3)	11.1 (3)	5.2 (2)	10.5 (4)
Loved ones missing^d^	–	12.1 (4)	48.1 (13)	70.3 (19)	21.1 (8)	28.9 (11)
Separation from loved ones^d^	–	–	29.6 (8)	–	47.3 (18)	–

*Note*: (−) not calculated. ^a^ Measured using the QII [16], scores range from −1 (“I do not agree”) to 1 (“I agree”). ^b^ Assessed with the stressor list of the CWE [18]. ^c^ *N* = 65 at baseline; the stressor list was not completed by stayers at baseline. ^d^ Compound score

### Study procedure

The follow-up assessment took place from June 2009 to March 2010, between 11 and 12 years after the baseline assessment and consequently 14 to 15 years after the end of the war in BiH. To identify former survey participants, earlier records were searched for participants’ addresses in 1998/1999. In the event of change of address or only names being available, home visits to the last known address, checks of phone book and death certificate records were carried out or requests submitted to municipal departments for refugees and displaced persons. All subjects provided written informed consent before participating in the study. Participants, who were under 18 at the baseline assessment, provided written informed consent themselves. The study protocol was approved by the Department of Psychology of the University of Munich, and the study was conducted in accordance with the Declaration of Helsinki.

Participants responded to a questionnaire package that was presented in face-to-face interviews by native speakers. All the questionnaires were adapted Bosnian versions that had undergone a thorough translation and back-translation process [15, 17]. Moreover, the interviewers helped complete the questionnaires when needed, which was often the case. All interviewers were trained in the assessment of relevant constructs and the use of the questionnaire measures, and were supervised by the second author. On average the interviews lasted 90 min. Participants received financial compensation of 20 convertible marks (about EUR 10).

### Measures

#### Sociodemographic and war-related characteristics

Sociodemographic information was obtained at both survey points, including age, gender, education, employment status, income, marital status, and mental health care use. War-related information (time spent in the war zone, material losses) was ascertained at baseline. Repatriation-related variables were assessed in displaced persons and returnees only using the *Questionnaire on Integration and Identification* (QII) [16] at baseline. The 12 items of the QII are scored on 3-point scales (“I do not agree” – “I agree”) and grouped into three categories: material satisfaction, identification with the home country, and community feeling [16].

#### Psychological distress

General psychological symptoms were assessed using the *Symptom Checklist-90-Revised* [21] at baseline, and its abbreviated version, the *Brief Symptom Inventory* (BSI) [22], at follow-up. Both versions are based on identical items and have been shown to present fairly equivalent psychometric properties [23, 24]. Hence, we focused our analyses on the BSI. The BSI is a widely used 53-item measure of subjective psychological distress experienced in the preceding seven days. All responses are scored on a 5-point scale from 0 (“not at all”) to 4 (“extremely”). The BSI’s nine subscales cover symptoms of clinically relevant psychological syndromes: somatization, obsessive-compulsive disorder, interpersonal sensitivity, depression, anxiety, phobic anxiety, paranoid ideation, and psychoticism. The Global Severity Index (GSI) is a measure of overall psychological distress and is calculated by summing up all nine subscales. Urbán and colleagues [24] proposed a bifactor model that supports reporting the nine subscales in addition to the rather sound GSI as outcome measures. In this study, Cronbach’s alpha of the GSI was .97 and .96 at baseline and follow-up, respectively.

#### Traumatic experiences

Traumatic events experienced during the war were measured using the 72-item *Checklist of War Related Experiences* (CWE) [18] at baseline. Forty-nine items of the CWE cover personally experienced and/or witnessed traumatic events (e.g., “During the war, did you stay in a cellar longer than 3 weeks without a break?”) that are either scored on 2-point or 3-point scales (0 = “no”, 1 = “yes/once”, 2 = “more than once”). A sum score of the total number of traumatic events was calculated (range: 0–98). Because of the CWE’s length, at follow-up, the Posttraumatic Diagnostic Scale [PDS; 25] was chosen to measure personally experienced and/or witnessed traumatic events during the war. The adapted PDS trauma list consisted of 13 yes/no-items (e.g., “I was tortured during the war.”). Traumatic events experienced before and after the war were assessed using adapted 13 yes/no item PDS trauma lists (e.g., “Before the war, I was sexually assaulted by a member of my family.”, “After the war, I suffered a life-threatening illness.”) [25]. Measures of the total number of traumatic events based on the PDS were calculated by adding up the items of the respective list. Scores ranged from 0 to 13.

#### Current stress factors

CWE items covering contemporary stressful living conditions [18] were used to assess current stress exposure. Twenty-three yes/no items were used at baseline and 12 at follow-up (e.g., “Are you entitled to a pension and/or welfare assistance but you do not receive it?”, “Is any other family member or close friend considered missing?”, Table 2). The types of stressful conditions were added up to create indices of the total number of current stressors at each survey point (range: 0–23 and 0–12).

### Data analysis

Differences between respondents and non-respondents were computed using χ^2^ tests, independent samples *t*-tests, and Mann-Whitney *U* tests. Differences between the three groups in sociodemographic characteristics and psychological symptoms were computed using χ^2^ tests and 3 × 2 analyses of variance (ANOVAs) with subsequent pairwise comparisons in the case of significant group or group x time interaction effects. These ANOVAs were followed by planned contrasts to test change on BSI subscales between baseline and follow-up for each group (see Additional file 1 for the ANOVA results). Sample differences in trauma and contemporary stress exposure were analyzed using one-way ANOVAs with group as the between-subjects factor and where appropriate, subsequent pairwise comparisons at each survey point. Two stepwise multiple regression analyses were calculated to investigate the associations between the GSI and potential predictor variables. Age, gender, education, and, for the follow-up model, the baseline dependent variable (GSI) were entered as control variables in the first step. The number of prewar traumatic events was included in the second step and the number of traumatic events during the war and group status were entered in the third step. Group status was recoded into two dummy variables, whereby displaced persons served as the reference category. Finally, postwar factors (i.e., numbers of current stressors and postwar traumatic events) were included in the model. The variance inflation factor (VIF) did not indicate serious multicollinearity among potential predictor variables (all VIFs were < 3). All tests were conducted two-tailed with α = .05. Across samples, there were missing values on five stressor-checklist items (in each case < 10%) which were not replaced. Data were analyzed with Stata version 13.0 (StataCorp, USA).

## Results

A total of 299 participants had been assessed at baseline in 1998/1999. In 2009/2010, minimal contact information could be obtained for 221 persons (74%). Out of these, 16 had died (5%), 67 could not be located (22%), and 38 declined to participate (13%). Health problems, lack of time (not related to health problems), and lack of interest were the most frequent reasons for not participating. For the 138 persons who were alive and could be located, the response rate was acceptable (71%). Consequently, 100 persons could be followed up in 2009/2010. Altogether three cases were excluded due to missing data, resulting in 198 non-respondents and a follow-up sample of 98 participants, consisting of 33 stayers, 27 former displaced persons, and 38 returnees.

### Sample characteristics

Drop-out analyses revealed only one significant difference: non-respondents to the follow-up assessment were less often married/in a long-term relationship (56%) than those who were followed up (68%; see Additional file 1). Baseline levels of BSI subscales and the GSI were similar between respondents and non-respondents (Additional file 1).

As shown in Table 1, the total sample was on average 36.5 years old (range: 16–67) at baseline. Participants had moderate educational attainment (about 11 years: 65%) and were mostly female (62%) and married (68%), and often unemployed (35%). At follow-up, participants were more often employed (51%, Table 1). Returnees achieved higher educational attainment than displaced persons (χ^2^ = 6.56, *df* = 2, *p* = .038). In contrast to stayers, returnees scored lower on somatization and obsessive-compulsive disorder subscales at baseline (*t*(95) = 2.48 and 3.11, *p* = .014 and .002, respectively) and higher on the hostility scale at follow-up (*t*(95) = − 2.05, *p* = .042). The three groups did not significantly differ in any other demographic characteristic or BSI score at both survey points.

### Trauma exposure

Lifetime exposure to traumatic events was high in all groups (Table 1). Moreover, as shown in Table 2, displaced persons and returnees had spent a considerable amount of time in the war zone, while stayers had been living in Sarajevo throughout the siege. At baseline, the majority of displaced persons and returnees identified with where they lived, but returnees mostly did not feel connected to the local community (Table 2). With regard to traumatic war events, stayers (baseline: *M* = 20.78, *SD* = 7.90; follow-up: *M* = 2.34, *SD* = 1.2) and displaced persons (baseline: *M* = 21.59, *SD* = 15.21; follow-up: *M* = 2.81, *SD* = 2.63) indicated greater exposure than returnees (baseline: *M* = 17.0, *SD* = 11.14; follow-up: *M* = 2.11, *SD* = 2.5) at baseline (*t*(95) = 1.99 and 2.24, *p* = .048 and .026, for stayers and displaced persons, respectively). Across samples, the most frequently reported events on the CWE were gunfire (74%), explosions (72%), deaths of family members (67%) and friends (70%), serious injuries of loved ones (65%), and conflict-related strains (e.g., poor access to food for a long time; 58%; see also [17, 18]). At follow-up, however, the three samples did not significantly differ in their exposure to traumatic war events. As different war stressor lists were employed at baseline (CWE, 49 items) and follow-up (adapted PDS, 13 items), the numbers at baseline and follow up cannot be compared directly. Furthermore, stayers reported experiencing more traumatic events before the war (*M* = 1.57, *SD* = 1.69) than displaced persons (*M* = 0.48, *SD* = 0.89, *t*(95) = 3.13, *p* = .002) and returnees (*M* = 0.89, *SD* = 2.50, *t*(95) = 2.13, *p* = .035) at baseline. At follow-up, the three groups did not significantly differ in the numbers of prewar traumatic events (stayers: *M* = 0.31, *SD* = 0.59; displaced: *M* = 0.33, *SD* = 0.73; returnees: *M* = 0.36, *SD* = 0.97). Moreover, the three samples did not significantly differ in their exposure to traumatic events that occurred after the war (stayers: *M* = 0.56, *SD* = 1.01; displaced persons: *M* = 0.48, *SD* = 1.05; returnees: *M* = 0.42, *SD* = 0.85).

### Exposure to current stressors

The exposure to current stress factors was high at both survey points (Table 1). Displaced persons (*M* = 4.0, *SD* = 2.46) and returnees (*M* = 3.27, *SD* = 2.26) did not significantly differ from each other in their baseline stress exposure. However, the formerly displaced (*M* = 3.01, *SD* = 1.35) reported more current stressors than stayers (*M* = 2.03, *SD* = 1.95, *t*(95) = − 2.91, *p* = .004) and returnees (*M* = 1.96, *SD* = 1.88, *t*(95) = 3.26, *p* = .001) at follow-up. Again, the numbers of reported current stressors at baseline and follow-up cannot be compared directly, as stressor lists of varying length were used. Table 2 lists reported types of stressors. Although similar stress factors were experienced frequently in all three groups (serious health problems, bad living and job conditions), distinct profiles emerged. At follow-up for instance, stayers and displaced persons often indicated poor access to medical care, while returnees and especially displaced persons frequently stated missing loved ones (Table 2).

### Change in psychological symptoms

**Fig. 1:**
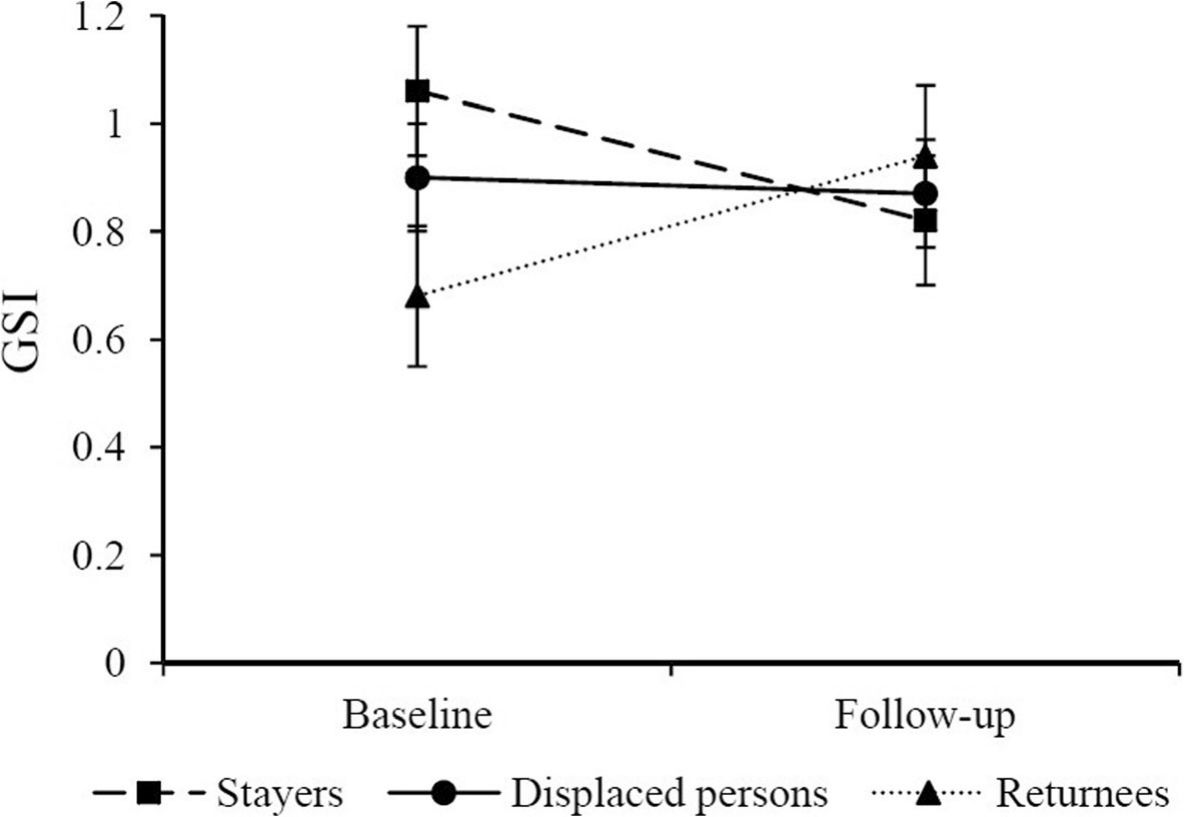
Means and *SEM*s of stayers (*n* = 33), displaced persons (*n* = 27), and returnees (*n* = 38) in the GSI (Global Severity Index) at baseline and follow-up

As shown in Fig. 1, the GSI decreased in stayers (*t*(95) = − 2.07, *p* = .042), did not change in displaced persons (*t*(95) = − 0.23, *p* = .815), and increased in returnees (*t*(95) = 2.35, *p* = .021) over time. Table 3 lists findings on psychological symptom trajectories of each group. Stayers showed significant decreases in symptoms of phobic anxiety, interpersonal sensitivity, and obsessive-compulsive disorder. Returnees, on the other hand, showed significantly increased somatization, obsessive-compulsive disorder, anxiety, hostility, paranoid ideation, and depression scores over time. Displaced persons did not present any significant changes.

**Table 3:** Means (*SD*) and change of psychological symptoms (BSI)

BSI	Stayers (*n* = 33)			Displaced (*n* = 27)			Returnee (*n* = 38)		
	Baseline	Follow-up	*t*	Baseline	Follow-up	*t*	Baseline	Follow-up	*t*
Somatization	1.26 (1.02)	1.10 (0.97)	−1.08	1.11 (0.95)	0.96 (0.80)	−0.88	0.70 (0.91)	1.08 (1.02)	2.65**
Obsessive-compulsive disorder	1.46 (1.18)	1.09 (0.86)	−2.23*	1.24 (1.06)	1.13 (0.83)	−0.61	0.75 (0.76)	1.16 (0.98)	2.62**
Interpersonal sensitivity	1.18 (0.83)	0.81 (0.74)	−2.43*	1.03 (1.0)	0.83 (0.69)	−1.17	0.78 (0.68)	0.79 (0.69)	0.09
Depression	0.98 (1.08)	0.73 (0.65)	−1.74	0.80 (0.86)	0.74 (0.52)	−0.38	0.64 (0.73)	0.99 (0.93)	2.58*
Anxiety	1.18 (1.15)	0.87 (0.74)	−1.68	1.11 (0.95)	1.07 (0.96)	−0.22	0.73 (0.78)	1.14 (1.01)	2.44*
Hostility	0.99 (0.71)	0.68 (0.61)	−1.85	0.82 (0.73)	0.90 (0.71)	0.41	0.67 (0.79)	1.06 (0.99)	2.51*
Phobic anxiety	0.85 (1.12)	0.46 (0.79)	−2.80**	0.70 (0.83)	0.51 (0.63)	−1.26	0.49 (0.68)	0.51 (0.50)	0.13
Paranoid ideation	1.28 (1.13)	1.19 (0.93)	−0.58	0.97 (0.76)	1.28 (0.95)	1.78	0.84 (0.75)	1.29 (0.95)	3.08**
Psychoticism	0.59 (0.69)	0.47 (0.64)	−0.89	0.39 (0.78)	0.48 (0.74)	0.61	0.48 (0.63)	0.55 (0.70)	0.53

*Note: BSI* = Brief Symptom Inventory (scores range from 0 to 4). Planned contrasts with *df* = 95 (scored as follow-up – baseline). * *p* < .05, ** *p* < .01

### Factors predicting general psychological distress

The results of the multiple regression analyses are shown in Table 4. At baseline, more prewar and war-related traumatic events were associated with a higher GSI. The total baseline model explained 28% of the GSI variance. In the follow-up model, the numbers of traumatic events during the war and postwar traumatic events, returnee status, and the number of current stressors emerged as positive predictors of the GSI. In total, 45% of the variance in the GSI was explained by the follow-up model.

**Table 4:** Hierarchical multiple regression analyses to predict general psychological distress (GSI)

Predictor	Baseline			Follow-up		
	β	*∆R*^2^/*∆F*	*R*^2^/*F*	β	*∆R*^2^/*∆F*	*R*^2^/*F*
Step 1						
Control variables^a^			.08/2.86*			.22/6.48***
Step 2						
Prewar traumatic events Married/long-term relationship	.25*	.10/11.18**	.18/5.18***	.02	.02/2.32	.24/5.73***
Step 3						
Traumatic events during the war	.23*			.21*		
Group status^b^						
Stayer	.24			.02		
Returnee	.01	.08/3.30*	.26/4.60***	.23*	.08/3.62*	.33/5.26***
Step 4						
Postwar traumatic events	–			.20*		
Current stressors	.19	.02/2.09	.28/4.33***	.33**	.12/9.10***	.45/6.84***

*Note: N* = 98. (−) not calculated. *BSI* = Brief Symptom Inventory. *GSI* = *BSI* Global Severity Index. ^a^ Control variables included age, gender, education, and, for follow-up model, baseline *GSI* (results omitted from the table). ^b^ Group status was represented as two dummy variables; displaced persons served as the reference group. * *p* < .05, ** *p* < .01, *** *p* < .001

## Discussion

### Main findings

This study examined the long-term mental health consequences of war exposure among civilians living in a post-conflict region. For this purpose, three samples of Sarajevo residents who had been assessed on average 3 years after the war were followed up 11 years later. We found that persons who had stayed in Sarajevo throughout the siege improved on the GSI, interpersonal sensitivity, phobic anxiety, and obsessive-compulsive disorder scores. Returnees displayed significant increases in the GSI and in symptoms of somatization, obsessive-compulsive disorder, paranoid ideation, hostility, anxiety and depression whereas displaced persons did not change on any BSI subscale. In 1998/1999, at baseline, total numbers of traumatic events experienced before and during the war predicted general psychological distress as measured using the GSI. At the 11-year follow-up, higher exposure to traumatic events during and after the war, returnee status, and more current stress factors were associated with higher GSI levels.

### Comparison with the literature

In the context of general mental distress, this study highlights the predictive value of cumulative traumatic experiences for mental health after war and displacement [6, 7, 9, 26]. In another Balkan sample, for instance, more traumatic war and postwar events were associated with higher rates of mental disorders between 5 and 15 years after war [8]. However, more prewar traumatic experiences were associated with higher distress at baseline but not at follow-up, while the number of traumatic events during the war emerged as a predictor at both survey points. These different associations might denote different psychological processes of coping with traumatization over time. Alternatively, the impact of prior traumatization on distress levels may decrease over time.

Research in the refugee field has documented the negative impact of stressful living conditions on mental health in the post-migration setting (e.g., lack of economic opportunities) [7, 9]. Most of this research is based on refugees living in high-income Western countries (e.g., [27–29]). All three groups of Sarajevo citizens reported relatively high exposure to current stressors. This comes as no surprise with regard to the postwar setting of this study which was characterized by a problematic process of reconstruction of institutions and infrastructure, and ongoing political conflict [19]. The number of current stressors emerged as the strongest predictor of the GSI at follow-up, whereas at baseline there was no significant association. The impact of recent war traumatization had perhaps prevailed in 1998/1999, which seems to support the notion that the impact of stressful living conditions increases over time [30]. This finding expands prior research in the region that had largely focused on single stress factors (e.g., health status, work conditions) [11, 14].

This study adds further findings on the long-term development of psychological symptoms. In all three samples, levels of BSI subscales were relatively high in comparison to another war-affected Balkan sample [31], but not as high as would be expected in clinical populations [23, 32]. In particular, paranoid ideation, obsessive-compulsive disorder, and somatization were more elevated than in the other Balkan sample, whereas depression scores were less elevated [31]. Even though the psychological symptom levels converged in the three samples at follow-up, earlier studies of conflict-affected populations have shown decreasing symptom levels over medium- to long-term follow-up periods [4, 14]. Our finding of differential symptom trajectories is more consistent with the varying somatic symptom trajectories of Croatian men over a 9-year postwar period [5]. The higher prewar and war-related trauma exposure of stayers could explain their relatively high baseline symptom levels and subsequent improvement. Previous research has revealed a tendency towards a reduced mental health risk in spite of high trauma exposure as more time since conflict elapses [9]. On the other hand, the influence of current living difficulties may increase over time [30], but stayers did not report more current stressors than the other two groups at follow-up. Moreover, there seemed to be no long-term changes in the displaced persons group, even though they indicated high war trauma and current stress exposure. With a sample size of 27, the power to detect changes in this group was rather low. The deterioration of returnees on several BSI subscales may reflect the particularly difficult social circumstances faced by this group. During the follow-up interviews, many returnees reported that they felt discriminated against by their fellow citizens who had not been externally displaced during the war. About half of the returnees had received financial incentives to return to BiH and/or were involuntarily repatriated in the late nineties [16]. Correspondingly, as assessed using the QII [16] at baseline, returnees mostly did not feel connected to their local community although they strongly identified with their home country. At follow-up, perceived discrimination may have increased feelings of isolation and insecurity and, consequently, psychological distress among returnees. Both discrimination and social isolation have been linked to worse mental health outcomes in refugees in high-income Western countries [28, 33, 34], lack of social support even in the longer term [35]. Future work might directly examine whether perceived discrimination and feelings of isolation are associated with increased mental distress in repatriated former refugees.

### Strengths and limitations

This study is the longest follow-up conducted on the course of general psychological symptoms among Bosnian war survivors. Three samples of Sarajevo citizens with different displacement experiences could be directly compared. Moreover, the instruments used in this study were presented by native speakers in face-to-face interviews. All questionnaires were adapted Bosnian versions that had undergone thorough translation procedures [15, 17]. In addition, the interviewers were trained, provided with specific instructions, and supervised on a regular basis.

Besides these strengths, several limitations need to be acknowledged. This study was observational so causality of any traumatic or stressful experiences cannot be established. Secondly, even though we went to considerable lengths to locate all baseline participants, only 138 persons from the total baseline sample (46%) could be located again. However, the post-war setting of this study needs to be taken into account. The study context was characterized by ongoing reconstruction and economic hardship as well as dynamic migratory patterns [19]. Overall, the location rate is about the same as the one reported for a shorter 6-year follow-up of conflict-affected Kosovars [14]. Furthermore, the response rate of the located persons was acceptable (71%) although the sample size of each group was rather small. Moreover, one might suspect some kind of systematic non-response, but participants and non-responders did not differ in any psychological, trauma, or demographic characteristics at baseline except for marital status. Still, we cannot determine how non-response influenced the results which hampers interpretation. Thirdly, trauma and current stressor lists of varying lengths were used at the two survey points. The baseline CWE was replaced by an adapted PDS trauma list at follow-up in order to reduce the stress of another lengthy trauma assessment. However, we cannot directly compare trauma reports at baseline and follow-up. Finally, we did not assess perceived discrimination and social support and were thus unable to examine their impact on mental distress. However, we accounted for a broad range of other contextual factors that could influence mental health outcomes (e.g., demographics, pre- and post-war trauma exposure, current stress factors, displacement experiences).

### Implications and conclusions

Our finding of a long-term negative impact of cumulative current stress exposure may be of interest to public health policies. In order to reduce long-term psychological distress after war and displacement, the broader material and social context needs to be taken into account [2, 26, 30]. It seems to be particularly important to allocate resources to improving material conditions (e.g., access to health care, work conditions). On a related note, it may be relevant to pay particular attention to the social conditions of former refugees in post-conflict settings. In this study, mental health outcomes of returnees deteriorated over time. Returnees had spent less than two years abroad during the war and had resettled in Sarajevo about 14 years prior to the follow-up assessment. Although refugee studies suggest an inverse relationship between length of displacement and mental health outcomes [26], our results show that poorer long-term outcomes also occur after relatively short displacement intervals. Moreover, these results highlight the importance of distinguishing between conflict-affected subpopulations, and the results may not generalize to refugees who settled in the West and did not return.

To conclude, this 11-year follow-up study is the longest follow-up conducted on the course and predictors of general mental health of Bosnians war survivors living in the post-conflict region. Over time, psychological symptom levels decreased in persons who had stayed in the war zone, persisted in former internally displaced persons, and increased in former refugees. The cumulative exposure to traumatic events and current stressors were associated with greater psychological distress about 14 years after the end of the war. Thus, the adverse mental health consequences of war traumatization and displacement are long lasting, and the impact of contemporary stressors seems to increase over the years. This highlights the need for economic and health sector reconstruction in order to promote mental health in post-conflict countries.

## Additional file

Additional file 1: **Table S1.** Baseline demographic and trauma characteristics of respondents and non-respondents to the follow-up assessment. **Table S2.** Means (*SD*) of baseline psychological symptoms of respondents and non-respondents to the follow-up assessment. **Table S3.** ANOVAs of psychological symptom scores (BSI) (DOCX 53 kb)
