# Supplementary material for: Long-term psychological distress of Bosnian war survivors: an 11-year follow-up of former displaced persons, returnees, and stayers
Source: BMC Psychiatry. 2019 Jan 3;19:1. doi: 10.1186/s12888-018-1996-0 (PMC6318963; doi:10.1186/s12888-018-1996-0)
Supplement: Supplementary file 1 — Table S1. Baseline demographic and trauma characteristics of respondents and non-respondents to the follow-up assessment. Table S2. Means (SD) of baseline psychological symptoms of respondents and non-respondents to the follow-up assessment. Table S3. ANOVAs of psychological symptom scores (BSI) (DOCX 53 kb) [file 12888_2018_1996_MOESM1_ESM.docx]

**Additional File 1**

Table S1: Baseline demographic and trauma characteristics of respondents and non-respondents to the follow-up assessment

| Characteristic | Respondents (*N*=98) | Non-respondents (*N*=198) | χ^2^ / *U* / *t* |
| --- | --- | --- | --- |
| Female, % (*n*) | 62.2 (61) | 51.0 (101) | 3.33 |
| Mean age (*SD*) | 36.5 (12.10) | 38.71 (14.25) | 1.30 |
| Education, % (*n*) |  |  | 2.51 |
| Primary | 20.4 (20) | 13.6 (27) |  |
| Secondary | 65.3 (64) | 70.2 (139) |  |
| Tertiary | 12.2 (12) | 15.1 (30) |  |
| Marital status, % (*n*) |  |  | 3.95* |
| Married/ long-term relationship | 68.4 (67) | 56.1 (111) |  |
| Single/ divorced/ separated/ widowed | 31.6 (31) | 43.4 (86) |  |
| Employment status, % (*n*) |  |  | 7.78 |
| Employed | 26.5 (26) | 26.7 (53) |  |
| Unemployed | 35.7 (35) | 34.8 (69) |  |
| Retired | 8.2 (8) | 14.1 (28) |  |
| In training/ education | 10.2 (10) | 14.1 (28) |  |
| Other | 19.3 (19) | 10.1 (20) |  |
| Monthly income, % (*n*) |  |  | 13.19 |
| No income | 16.2 (16) | 13.1 (26) |  |
| < 500 KM | 36.7 (36) | 41.4 (82) |  |
| 500 – 1000 KM | 6.1 (6) | 5.6 (11) |  |
| > 1000 KM | 4.0 (4) | 2.0 (4) |  |
| Number of traumatic events, mean (*SD*) |  |  |  |
| Prewar traumatic events | 1.01 (1.93) | 0.71 (1.15) | 9163.5 |
| Traumatic events during the war | 19.54 (11.58)^a^ | 18.94 (11.58) | 9345.5 |
| Postwar traumatic events | – | – |  |
| Number of current stressors, mean (*SD*) | 2.48 (2.47)^c^ | 2.16 (2.33) | 9003.5 |

Note: KM = “convertible Marks”. ^a^ Traumatic events assessed using the CWE (Rosner et al. [18]; range: 0–98). ^b^ Adapted trauma list of the PDS (Foa et al. [25]; range: 0–13). ^c^ 23-item stressor list based on the CWE (range: 0–23), *N* = 65 as the checklist was not completed by stayers. ^d^ 12-item stressor list based on the CWE (range: 0–12). * *p* < .05.

Table S2: Means (*SD*) of baseline psychological symptoms of respondents and non-respondents to the follow-up assessment

| BSI | Respondents (*N*=98) | Non-respondents (*N*=198) | *t* |
| --- | --- | --- | --- |
| Somatization | 1.0 (0.98) | 0.90 (0.97) | 0.78 |
| Obsessive-compulsive disorder | 1.13 (1.03) | 0.98 (0.91) | 1.18 |
| Interpersonal sensitivity | 0.98 (0.83) | 0.96 (0.81) | 0.17 |
| Depression | 0.80 (0.89) | 0.79 (0.78) | 0.04 |
| Anxiety | 0.98 (0.97) | 0.92 (0.93) | 0.53 |
| Hostility | 0.82 (0.75) | 0.76 (0.77) | 0.60 |
| Phobic anxiety | 0.67 (0.89) | 0.56 (0.72) | 1.06 |
| Paranoid ideation | 1.02 (0.90) | 0.98 (0.88) | 0.41 |
| Psychoticism | 0.49 (0.69) | 0.38 (0.55) | 1.40 |
| GSI | 0.87 (0.75) | 0.80 (0.67) | 0.85 |

Note: BSI = Brief Symptom Inventory (scales range from 0 to 4). GSI = BSI Global Severity Index (range: 0–4). *T*-tests scored as respondents – non-respondents.

Table S3: ANOVAs of psychological symptom scores (BSI)

|  | Group^a^ | | Time^b^ | | Group x Time^a^ | |
| --- | --- | --- | --- | --- | --- | --- |
| BSI | *F* | η^2^ | *F* | η^2^ | *F* | η^2^ |
| Somatization | 1.02 | .02 | 0.06 | .00 | 4.31* | .08 |
| Obsessive-compulsive disorder | 1.41 | .03 | 0.07 | .00 | 6.11** | .11 |
| Interpersonal sensitivity | 1.03 | .02 | 4.28* | .05 | 1.71 | .03 |
| Depression | 0.10 | .00 | 0.02 | .00 | 4.84** | .09 |
| Anxiety | 0.31 | .01 | 0.04 | .00 | 4.32* | .08 |
| Hostility | 0.02 | .00 | 0.28 | .00 | 4.70* | .09 |
| Phobic anxiety | 0.59 | .01 | 5.37* | .06 | 2.30 | .05 |
| Paranoid ideation | 0.38 | .01 | 5.88* | .07 | 3.33* | .06 |
| Psychoticism | 0.26 | .00 | 0.03 | .00 | 0.70 | .01 |
| GSI | 0.39 | .01 | 0.01 | .00 | 4.91** | .09 |

*Note:* *N* = 98. BSI = Brief Symptom Inventory (scales range from 0 to 4). GSI = BSI Global Severity Index (range: 0–4). ^a^ df = 2/95. ^b^ df = 1/95. * p < .05, ** p < .01.
